# Bis[2,3-bis­(pyridin-2-yl)pyrazine-κ^2^ *N* ^2^,*N* ^3^]palladium(II) dinitrate aceto­nitrile monosolvate

**DOI:** 10.1107/S241431462100153X

**Published:** 2021-02-12

**Authors:** Kwang Ha

**Affiliations:** a Chonnam National University, School of Chemical Engineering, Research Institute of Catalysis, Gwangju, Republic of Korea; Benemérita Universidad Autónoma de Puebla, México

**Keywords:** crystal structure, palladium(II) complex, square-planar structure, 2,3-di-2-pyridyl­pyrazine

## Abstract

The title compound, [Pd(C_14_H_10_N_4_)_2_](NO_3_)_2_·CH_3_CN, consists of a cationic Pd^II^ complex, two anions and one lattice solvent mol­ecule, all in general positions. In the complex, the Pd^II^ cation is four-coordinated in a slightly distorted square-planar geometry defined by the four N atoms of two bidentate 2,3-di-2-pyridyl­pyrazine ligands. The complex, anions and solvent mol­ecule are linked by weak C—H⋯O inter­molecular hydrogen bonds. In the crystal, the complex mol­ecules are stacked in columns along the *a* axis.

## Abstract 2

In the title complex, the central Pd^II^ ion has an N_4_ square-planar coordination geometry defined by the four N atoms of two bidentate 2,3-di-2-pyridyl­pyrazine ligands.

## Graphical abstract

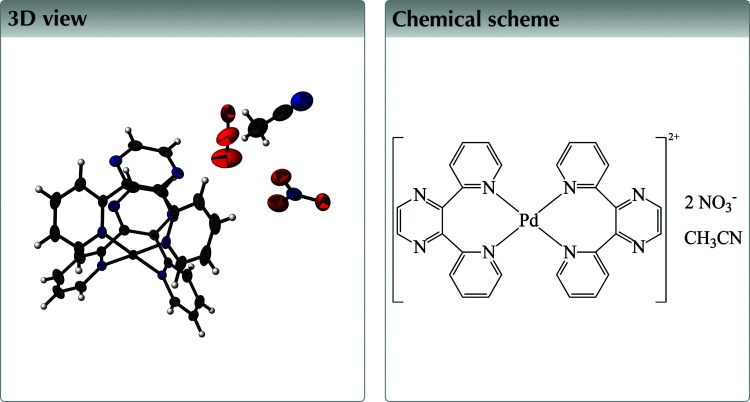

## Structure description

With reference to the title compound, [Pd(dpp)_2_](NO_3_)_2_·CH_3_CN (dpp = 2,3-di-2-pyridyl­pyrazine), the crystal structures of related dpp-Pd^II^ complexes [Pd*X*_2_(dpp)] have been determined previously [*X* = Cl (Ha, 2011 **a* ▸,b* ▸); *X* = Br (Ha, 2011*c* ▸); *X* = I (Ha, 2011*d* ▸); *X* = SCN (Ha, 2012 ▸)], and a heterometallic complex has been also reported, namely [Ru(bipy)_2_(μ_2_-dpp)PdCl_2_](PF_6_)_2_, where bipy is 2,2′-bi­pyridine (Yam *et al.*, 1994 ▸).

**Figure 1:**
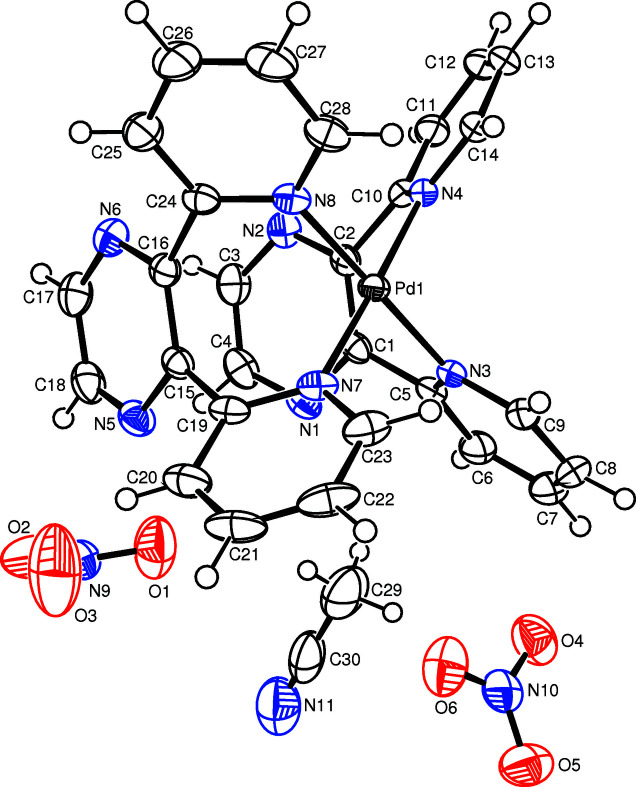
The mol­ecular structure of the title compound showing the atom labelling and displacement ellipsoids drawn at the 50% probability level for non-H atoms.

The title compound consists of a cationic Pd^II^ complex [Pd(dpp)_2_]^2+^, two NO_3_^−^ anions and one solvent CH_3_CN mol­ecule (Fig. 1 ▸). In the complex, the central Pd^II^ cation is four-coordinated in a slightly distorted square-planar geometry defined by the pyridyl N3, N4, N7 and N8 atoms of the two bidentate dpp ligands. The tight N—Pd—N chelating angles of N3—Pd1—N4 = 86.99 (7)° and N7—Pd1—N8 = 85.98 (7)° contribute to the distortion of the square-plane. The Pd—N bond lengths are almost equal [2.0170 (18) to 2.0286 (19) Å]. In the crystal, the pyridine rings are considerably inclined to the least-squares plane of the [PdN_4_] unit [maximum deviation = 0.0204 (7) Å], with dihedral angles of 70.56 (7) (ring N3⋯C9), 67.63 (6) (ring N4⋯C14), 71.32 (6) (ring N7⋯C23) and 71.64 (7)° (ring N8⋯C28). The nearly planar pyrazine rings [maximum deviation = 0.027 (2) Å] are fairly perpendicular to the [PdN_4_] unit plane, with dihedral angles of 82.07 (7) (ring N1⋯C4) and 84.20 (7)° (ring N5⋯C18).

**Figure 2:**
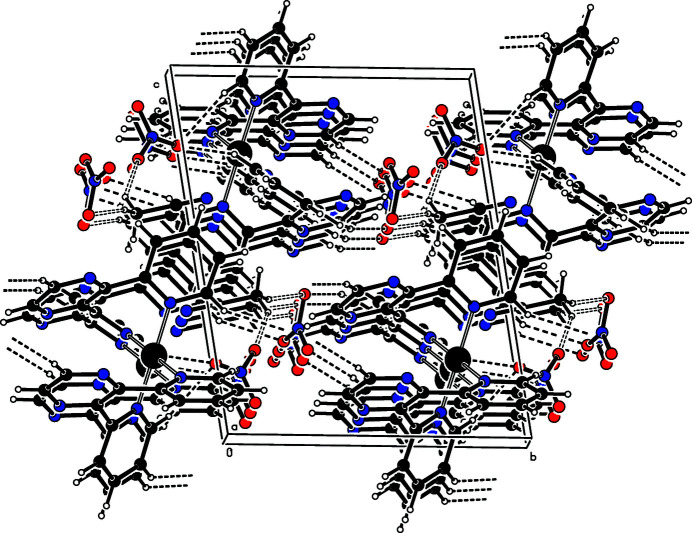
The packing in the crystal structure of the title compound, viewed approximately along the *a* axis. Hydrogen-bonding inter­actions are drawn as dashed lines.

In the crystal structure (Fig. 2 ▸), the complex, anions and solvent mol­ecules form inter­molecular weak C—H⋯O hydrogen bonds (Table 1 ▸). The complex mol­ecules are stacked in columns along the *a* axis. In the columns, numerous intra- and inter­molecular π–π inter­actions between adjacent six-membered rings are present. For *Cg*1 (the centroid of ring N4⋯C14) and *Cg*1^i^ [symmetry code: (i) −*x* + 2, −*y* + 2, −*z* + 1), the centroid-to-centroid separation is 3.688 (2) Å and the planes are parallel and shifted by 1.683 Å.

## Synthesis and crystallization

To a solution of 2,3-di-2-pyridyl­pyrazine (0.303 g, 1.293 mmol) in acetone (30 ml) was added Pd(NO_3_)_2_·2H_2_O (0.170 g, 0.637 mmol) and stirred for 1 h at room temperature. The formed precipitate was recrystallized from MeOH/ether, washed with ether, and dried under vacuum, to give a white powder (0.378 g). Crystals suitable for X-ray analysis were obtained by slow evaporation of a MeOH/CH_3_CN solution, at room temperature.

## Refinement

Crystal data, data collection and structure refinement details are summarized in Table 2 ▸. The highest peak (0.89 e Å^−3^) and the deepest hole (−0.49 e Å^−3^) in the last difference-Fourier map are located 1.02 and 0.97 Å, respectively, from atom O3.

## Supplementary Material

Crystal structure: contains datablock(s) I. DOI: 10.1107/S241431462100153X/bh4059sup1.cif

Structure factors: contains datablock(s) I. DOI: 10.1107/S241431462100153X/bh4059Isup2.hkl

CCDC reference: 2062092

Additional supporting information:  crystallographic information; 3D view; checkCIF report

## Figures and Tables

**Table 1:** Hydrogen-bond geometry (Å, °)

*D*—H⋯*A*	*D*—H	H⋯*A*	*D*⋯*A*	*D*—H⋯*A*
C3—H3⋯O4^i^	0.94	2.43	3.344 (3)	164
C4—H4⋯O1	0.94	2.51	3.330 (4)	146
C9—H9⋯O2^ii^	0.94	2.40	3.113 (4)	133
C14—H14⋯O5^iii^	0.94	2.43	3.257 (3)	147
C17—H17⋯O6^i^	0.94	2.43	3.343 (3)	164
C22—H22⋯O3^iv^	0.94	2.45	3.273 (5)	146
C23—H23⋯O2^ii^	0.94	2.54	3.275 (4)	136
C29—H29*A*⋯O1	0.97	2.58	3.405 (5)	144
C29—H29*C*⋯O4	0.97	2.51	3.459 (4)	167

Symmetry codes: (i) 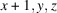 ; (ii) 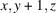 ; (iii) 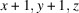 ; (iv) 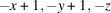 .

**Table 2:** Experimental details

Crystal data	
Chemical formula	[Pd(C_14_H_10_N_4_)_2_](NO_3_)_2_·C_2_H_3_N
*M*_r_	739.99
Crystal system, space group	Triclinic, *P* 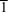
Temperature (K)	223
*a*, *b*, *c* (Å)	10.2013 (4), 11.6159 (5), 14.2762 (6)
α, β, γ (°)	90.9753 (16), 109.2479 (14), 109.9435 (14)
*V* (Å^3^)	1485.28 (11)
*Z*	2
Radiation type	Mo *K*α
μ (mm^−1^)	0.69
Crystal size (mm)	0.22 × 0.15 × 0.09

Data collection	
Diffractometer	Bruker *APEX2*, PHOTON 100 detector
Absorption correction	Multi-scan (*SADABS*; Bruker, 2016 ▸)
*T*_min_, *T*_max_	0.699, 0.745
No. of measured, independent and observed [*I* > 2σ(*I*)] reflections	38859, 5889, 5399
*R*_int_	0.032
(sin θ/λ)_max_ (Å^−1^)	0.619

Refinement	
*R*[*F* ^2^ > 2σ(*F* ^2^)], *wR*(*F* ^2^), *S*	0.027, 0.066, 1.06
No. of reflections	5889
No. of parameters	434
H-atom treatment	H-atom parameters constrained
Δρ_max_, Δρ_min_ (e Å^−3^)	0.89, −0.49

Computer programs: *APEX2* and *SAINT* (Bruker, 2016 ▸), *SHELXT2014/7* (Sheldrick, 2015*a* ▸), *SHELXL2014/7* (Sheldrick, 2015*b* ▸), *ORTEP-3 for Windows* (Farrugia, 2012 ▸) and *PLATON* (Spek, 2020 ▸).

## full crystallographic data

### Crystal data

**Table:**

[Pd(C_14_H_10_N_4_)_2_](NO_3_)_2_·C_2_H_3_N	*Z* = 2
*M**_r_* = 739.99	*F*(000) = 748
Triclinic, *P*1	*D*_x_ = 1.655 Mg m^−^^3^
*a* = 10.2013 (4) Å	Mo *K*α radiation, λ = 0.71073 Å
*b* = 11.6159 (5) Å	Cell parameters from 9802 reflections
*c* = 14.2762 (6) Å	θ = 2.3–28.3°
α = 90.9753 (16)°	µ = 0.69 mm^−^^1^
β = 109.2479 (14)°	*T* = 223 K
γ = 109.9435 (14)°	Block, colorless
*V* = 1485.28 (11) Å^3^	0.22 × 0.15 × 0.09 mm

### Data collection

**Table:**

Bruker APEX2, PHOTON 100 detector diffractometer	5399 reflections with *I* > 2σ(*I*)
Radiation source: sealed tube	*R*_int_ = 0.032
φ and ω scans	θ_max_ = 26.1°, θ_min_ = 2.2°
Absorption correction: multi-scan (SADABS; Bruker, 2016)	*h* = −12→12
*T*_min_ = 0.699, *T*_max_ = 0.745	*k* = −14→14
38859 measured reflections	*l* = −17→17
5889 independent reflections	

### Refinement

**Table:**

Refinement on *F*^2^	Primary atom site location: structure-invariant direct methods
Least-squares matrix: full	Secondary atom site location: difference Fourier map
*R*[*F*^2^ > 2σ(*F*^2^)] = 0.027	Hydrogen site location: inferred from neighbouring sites
*wR*(*F*^2^) = 0.066	H-atom parameters constrained
*S* = 1.06	*w* = 1/[σ^2^(*F*_o_^2^) + (0.030*P*)^2^ + 1.3932*P*] where *P* = (*F*_o_^2^ + 2*F*_c_^2^)/3
5889 reflections	(Δ/σ)_max_ = 0.001
434 parameters	Δρ_max_ = 0.89 e Å^−^^3^
0 restraints	Δρ_min_ = −0.49 e Å^−^^3^

### Special details

**Table:**

Refinement. Hydrogen atoms bonded to C atoms were positioned geometrically and allowed to ride on their parent atoms: C—H = 0.94 or 0.97 Å, with isotropic displacements *U*_iso_(H) = 1.2*U*_eq_(C) or 1.5*U*_eq_(methyl C).

### Fractional atomic coordinates and isotropic or equivalent isotropic displacement parameters (Å^2^)

**Table:**

	*x*	*y*	*z*	*U*_iso_*/*U*_eq_	
Pd1	0.85573 (2)	0.80002 (2)	0.21420 (2)	0.02031 (6)	
N1	0.8010 (2)	0.47002 (17)	0.32663 (14)	0.0296 (4)	
N2	1.1007 (2)	0.62333 (18)	0.41615 (14)	0.0300 (4)	
N3	0.70063 (19)	0.72337 (16)	0.27734 (14)	0.0243 (4)	
N4	1.00126 (19)	0.87763 (16)	0.35473 (13)	0.0226 (4)	
N5	0.8194 (2)	0.46396 (17)	0.07565 (15)	0.0318 (4)	
N6	1.1185 (2)	0.61800 (19)	0.15483 (15)	0.0316 (4)	
N7	0.71597 (19)	0.71968 (16)	0.07375 (13)	0.0249 (4)	
N8	1.01151 (19)	0.87359 (16)	0.15207 (13)	0.0243 (4)	
C1	0.8420 (2)	0.59294 (19)	0.34974 (15)	0.0228 (4)	
C2	0.9924 (2)	0.6705 (2)	0.39218 (15)	0.0232 (4)	
C3	1.0584 (3)	0.5018 (2)	0.39210 (18)	0.0342 (5)	
H3	1.1317	0.4662	0.4075	0.041*	
C4	0.9105 (3)	0.4261 (2)	0.34529 (18)	0.0336 (5)	
H4	0.8864	0.3415	0.3261	0.040*	
C5	0.7133 (2)	0.6331 (2)	0.33404 (16)	0.0259 (5)	
C6	0.6064 (3)	0.5744 (2)	0.3748 (2)	0.0380 (6)	
H6	0.6146	0.5096	0.4121	0.046*	
C7	0.4865 (3)	0.6125 (3)	0.3599 (2)	0.0478 (7)	
H7	0.4132	0.5743	0.3874	0.057*	
C8	0.4767 (3)	0.7063 (3)	0.3047 (2)	0.0470 (7)	
H8	0.3977	0.7344	0.2950	0.056*	
C9	0.5843 (2)	0.7592 (2)	0.2632 (2)	0.0350 (6)	
H9	0.5757	0.8223	0.2239	0.042*	
C10	1.0482 (2)	0.8070 (2)	0.42188 (16)	0.0233 (4)	
C11	1.1519 (2)	0.8600 (2)	0.51676 (17)	0.0308 (5)	
H11	1.1821	0.8097	0.5635	0.037*	
C12	1.2106 (3)	0.9870 (2)	0.54251 (19)	0.0364 (6)	
H12	1.2824	1.0240	0.6064	0.044*	
C13	1.1629 (3)	1.0586 (2)	0.47366 (19)	0.0342 (5)	
H13	1.2022	1.1453	0.4895	0.041*	
C14	1.0563 (2)	1.0014 (2)	0.38061 (18)	0.0287 (5)	
H14	1.0213	1.0503	0.3342	0.034*	
C15	0.8605 (2)	0.58748 (19)	0.07894 (15)	0.0237 (4)	
C16	1.0115 (2)	0.6651 (2)	0.11952 (15)	0.0238 (4)	
C17	1.0748 (3)	0.4960 (2)	0.15386 (19)	0.0367 (6)	
H17	1.1473	0.4608	0.1803	0.044*	
C18	0.9262 (3)	0.4196 (2)	0.11519 (19)	0.0359 (6)	
H18	0.9000	0.3341	0.1169	0.043*	
C19	0.7352 (2)	0.6281 (2)	0.02701 (16)	0.0255 (5)	
C20	0.6387 (3)	0.5709 (2)	−0.06908 (18)	0.0372 (6)	
H20	0.6503	0.5048	−0.1000	0.045*	
C21	0.5258 (3)	0.6114 (3)	−0.11883 (19)	0.0451 (7)	
H21	0.4610	0.5743	−0.1844	0.054*	
C22	0.5088 (3)	0.7067 (3)	−0.0719 (2)	0.0423 (7)	
H22	0.4329	0.7360	−0.1050	0.051*	
C23	0.6045 (2)	0.7585 (2)	0.02450 (19)	0.0333 (5)	
H23	0.5919	0.8228	0.0570	0.040*	
C24	1.0708 (2)	0.8015 (2)	0.11849 (16)	0.0250 (4)	
C25	1.1870 (3)	0.8527 (2)	0.08430 (19)	0.0367 (6)	
H25	1.2285	0.8019	0.0619	0.044*	
C26	1.2419 (3)	0.9791 (3)	0.0832 (2)	0.0444 (7)	
H26	1.3218	1.0151	0.0609	0.053*	
C27	1.1782 (3)	1.0513 (2)	0.1153 (2)	0.0409 (6)	
H27	1.2132	1.1372	0.1143	0.049*	
C28	1.0624 (3)	0.9964 (2)	0.14890 (18)	0.0329 (5)	
H28	1.0180	1.0456	0.1701	0.039*	
N9	0.7053 (3)	0.0811 (2)	0.17671 (19)	0.0425 (5)	
O1	0.6818 (3)	0.1389 (3)	0.23751 (18)	0.0810 (8)	
O2	0.7579 (3)	0.0012 (2)	0.1979 (3)	0.1035 (12)	
O3	0.6834 (5)	0.1134 (4)	0.0945 (2)	0.1297 (15)	
N10	0.2051 (3)	0.3037 (2)	0.3109 (2)	0.0463 (6)	
O4	0.2775 (3)	0.3406 (2)	0.40190 (18)	0.0636 (6)	
O5	0.0679 (2)	0.25210 (19)	0.2792 (2)	0.0688 (7)	
O6	0.2740 (3)	0.3221 (2)	0.2514 (2)	0.0695 (7)	
N11	0.3636 (4)	−0.0592 (4)	0.3507 (3)	0.0822 (10)	
C29	0.4999 (4)	0.1768 (4)	0.3903 (4)	0.0813 (12)	
H29A	0.5525	0.2046	0.3445	0.122*	
H29B	0.5707	0.1988	0.4587	0.122*	
H29C	0.4278	0.2163	0.3828	0.122*	
C30	0.4232 (4)	0.0439 (4)	0.3679 (3)	0.0623 (9)	

### Atomic displacement parameters (Å^2^)

**Table:**

	*U*^11^	*U*^22^	*U*^33^	*U*^12^	*U*^13^	*U*^23^
Pd1	0.01659 (9)	0.01646 (9)	0.02448 (9)	0.00547 (6)	0.00380 (6)	0.00146 (6)
N1	0.0380 (11)	0.0216 (9)	0.0258 (10)	0.0080 (8)	0.0102 (8)	0.0027 (7)
N2	0.0320 (10)	0.0327 (11)	0.0268 (10)	0.0149 (9)	0.0090 (8)	0.0052 (8)
N3	0.0180 (8)	0.0207 (9)	0.0288 (9)	0.0041 (7)	0.0052 (7)	−0.0062 (7)
N4	0.0180 (8)	0.0204 (9)	0.0275 (9)	0.0045 (7)	0.0087 (7)	−0.0026 (7)
N5	0.0367 (11)	0.0227 (10)	0.0338 (11)	0.0063 (8)	0.0145 (9)	0.0021 (8)
N6	0.0303 (10)	0.0342 (11)	0.0344 (11)	0.0151 (9)	0.0132 (9)	0.0068 (9)
N7	0.0195 (9)	0.0219 (9)	0.0266 (9)	0.0043 (7)	0.0034 (7)	0.0074 (7)
N8	0.0222 (9)	0.0218 (9)	0.0230 (9)	0.0048 (7)	0.0041 (7)	0.0042 (7)
C1	0.0288 (11)	0.0206 (10)	0.0178 (10)	0.0072 (9)	0.0088 (8)	0.0019 (8)
C2	0.0267 (11)	0.0248 (11)	0.0172 (10)	0.0080 (9)	0.0083 (8)	0.0019 (8)
C3	0.0437 (14)	0.0345 (13)	0.0334 (13)	0.0228 (11)	0.0158 (11)	0.0110 (10)
C4	0.0504 (15)	0.0231 (11)	0.0302 (12)	0.0167 (11)	0.0144 (11)	0.0075 (9)
C5	0.0227 (10)	0.0209 (10)	0.0273 (11)	0.0011 (8)	0.0079 (9)	−0.0064 (9)
C6	0.0346 (13)	0.0308 (13)	0.0443 (15)	0.0004 (10)	0.0210 (11)	−0.0030 (11)
C7	0.0325 (14)	0.0420 (16)	0.0607 (18)	−0.0029 (12)	0.0246 (13)	−0.0120 (14)
C8	0.0197 (12)	0.0479 (16)	0.0647 (19)	0.0058 (11)	0.0126 (12)	−0.0196 (14)
C9	0.0212 (11)	0.0314 (13)	0.0450 (14)	0.0087 (10)	0.0042 (10)	−0.0107 (11)
C10	0.0178 (10)	0.0246 (11)	0.0265 (11)	0.0056 (8)	0.0090 (8)	−0.0013 (9)
C11	0.0244 (11)	0.0354 (13)	0.0266 (11)	0.0074 (10)	0.0058 (9)	−0.0034 (10)
C12	0.0239 (11)	0.0386 (14)	0.0344 (13)	0.0028 (10)	0.0050 (10)	−0.0142 (11)
C13	0.0261 (12)	0.0242 (11)	0.0454 (14)	−0.0001 (9)	0.0146 (11)	−0.0131 (10)
C14	0.0247 (11)	0.0201 (11)	0.0405 (13)	0.0044 (9)	0.0149 (10)	−0.0029 (9)
C15	0.0280 (11)	0.0210 (10)	0.0204 (10)	0.0057 (9)	0.0102 (9)	0.0017 (8)
C16	0.0268 (11)	0.0255 (11)	0.0194 (10)	0.0083 (9)	0.0103 (9)	0.0033 (8)
C17	0.0438 (14)	0.0375 (14)	0.0409 (14)	0.0238 (12)	0.0208 (12)	0.0103 (11)
C18	0.0488 (15)	0.0243 (12)	0.0417 (14)	0.0159 (11)	0.0224 (12)	0.0051 (10)
C19	0.0217 (10)	0.0218 (10)	0.0234 (11)	−0.0001 (8)	0.0047 (9)	0.0039 (8)
C20	0.0319 (13)	0.0374 (14)	0.0262 (12)	−0.0010 (11)	0.0048 (10)	−0.0002 (10)
C21	0.0296 (13)	0.0485 (16)	0.0281 (13)	−0.0054 (12)	−0.0055 (10)	0.0073 (12)
C22	0.0210 (12)	0.0460 (15)	0.0435 (15)	0.0036 (11)	−0.0006 (11)	0.0237 (13)
C23	0.0238 (11)	0.0300 (12)	0.0419 (14)	0.0092 (10)	0.0067 (10)	0.0152 (10)
C24	0.0236 (10)	0.0242 (11)	0.0206 (10)	0.0035 (9)	0.0052 (8)	0.0024 (8)
C25	0.0375 (13)	0.0353 (13)	0.0361 (13)	0.0060 (11)	0.0195 (11)	0.0033 (11)
C26	0.0458 (15)	0.0380 (14)	0.0452 (15)	−0.0009 (12)	0.0280 (13)	0.0082 (12)
C27	0.0478 (15)	0.0254 (12)	0.0403 (14)	0.0003 (11)	0.0176 (12)	0.0103 (11)
C28	0.0362 (13)	0.0222 (11)	0.0325 (12)	0.0061 (10)	0.0074 (10)	0.0061 (9)
N9	0.0446 (13)	0.0276 (11)	0.0576 (15)	0.0164 (10)	0.0179 (11)	0.0038 (10)
O1	0.0879 (19)	0.124 (2)	0.0508 (14)	0.0659 (18)	0.0220 (13)	0.0027 (15)
O2	0.0523 (14)	0.0291 (11)	0.209 (4)	0.0242 (11)	0.0119 (18)	0.0110 (16)
O3	0.202 (4)	0.184 (4)	0.071 (2)	0.125 (4)	0.075 (2)	0.043 (2)
N10	0.0505 (15)	0.0224 (11)	0.0667 (17)	0.0219 (10)	0.0132 (13)	0.0075 (11)
O4	0.0732 (16)	0.0563 (14)	0.0582 (14)	0.0345 (12)	0.0090 (12)	−0.0004 (11)
O5	0.0470 (13)	0.0366 (11)	0.110 (2)	0.0152 (10)	0.0123 (13)	0.0097 (12)
O6	0.0829 (17)	0.0708 (16)	0.0788 (17)	0.0431 (14)	0.0430 (15)	0.0216 (13)
N11	0.093 (3)	0.092 (3)	0.079 (2)	0.052 (2)	0.034 (2)	0.008 (2)
C29	0.060 (2)	0.084 (3)	0.115 (3)	0.046 (2)	0.030 (2)	0.015 (2)
C30	0.061 (2)	0.087 (3)	0.059 (2)	0.052 (2)	0.0209 (17)	0.008 (2)

### Geometric parameters (Å, º)

**Table:**

Pd1—N7	2.0170 (18)	C12—H12	0.9400
Pd1—N8	2.0178 (18)	C13—C14	1.383 (3)
Pd1—N4	2.0257 (17)	C13—H13	0.9400
Pd1—N3	2.0286 (19)	C14—H14	0.9400
N1—C4	1.333 (3)	C15—C16	1.403 (3)
N1—C1	1.345 (3)	C15—C19	1.485 (3)
N2—C3	1.329 (3)	C16—C24	1.493 (3)
N2—C2	1.344 (3)	C17—C18	1.380 (4)
N3—C9	1.344 (3)	C17—H17	0.9400
N3—C5	1.351 (3)	C18—H18	0.9400
N4—C14	1.347 (3)	C19—C20	1.388 (3)
N4—C10	1.347 (3)	C20—C21	1.376 (4)
N5—C18	1.325 (3)	C20—H20	0.9400
N5—C15	1.346 (3)	C21—C22	1.373 (4)
N6—C17	1.331 (3)	C21—H21	0.9400
N6—C16	1.341 (3)	C22—C23	1.376 (4)
N7—C19	1.349 (3)	C22—H22	0.9400
N7—C23	1.350 (3)	C23—H23	0.9400
N8—C24	1.349 (3)	C24—C25	1.381 (3)
N8—C28	1.349 (3)	C25—C26	1.384 (4)
C1—C2	1.400 (3)	C25—H25	0.9400
C1—C5	1.490 (3)	C26—C27	1.375 (4)
C2—C10	1.490 (3)	C26—H26	0.9400
C3—C4	1.381 (4)	C27—C28	1.380 (4)
C3—H3	0.9400	C27—H27	0.9400
C4—H4	0.9400	C28—H28	0.9400
C5—C6	1.385 (3)	N9—O3	1.211 (4)
C6—C7	1.392 (4)	N9—O2	1.218 (3)
C6—H6	0.9400	N9—O1	1.219 (3)
C7—C8	1.367 (4)	N10—O5	1.235 (3)
C7—H7	0.9400	N10—O4	1.245 (3)
C8—C9	1.383 (4)	N10—O6	1.248 (3)
C8—H8	0.9400	N11—C30	1.123 (5)
C9—H9	0.9400	C29—C30	1.446 (6)
C10—C11	1.384 (3)	C29—H29A	0.9700
C11—C12	1.380 (3)	C29—H29B	0.9700
C11—H11	0.9400	C29—H29C	0.9700
C12—C13	1.372 (4)		
			
N7—Pd1—N8	85.98 (7)	C14—C13—H13	120.4
N7—Pd1—N4	177.88 (7)	N4—C14—C13	121.8 (2)
N8—Pd1—N4	92.86 (7)	N4—C14—H14	119.1
N7—Pd1—N3	94.14 (7)	C13—C14—H14	119.1
N8—Pd1—N3	178.89 (7)	N5—C15—C16	120.7 (2)
N4—Pd1—N3	86.99 (7)	N5—C15—C19	113.75 (19)
C4—N1—C1	116.7 (2)	C16—C15—C19	125.20 (19)
C3—N2—C2	116.9 (2)	N6—C16—C15	120.9 (2)
C9—N3—C5	119.0 (2)	N6—C16—C24	113.55 (19)
C9—N3—Pd1	121.60 (17)	C15—C16—C24	125.26 (19)
C5—N3—Pd1	119.40 (14)	N6—C17—C18	121.9 (2)
C14—N4—C10	119.34 (19)	N6—C17—H17	119.0
C14—N4—Pd1	119.98 (15)	C18—C17—H17	119.0
C10—N4—Pd1	120.63 (14)	N5—C18—C17	121.6 (2)
C18—N5—C15	117.5 (2)	N5—C18—H18	119.2
C17—N6—C16	117.2 (2)	C17—C18—H18	119.2
C19—N7—C23	119.2 (2)	N7—C19—C20	120.7 (2)
C19—N7—Pd1	120.11 (14)	N7—C19—C15	119.79 (18)
C23—N7—Pd1	120.68 (17)	C20—C19—C15	119.5 (2)
C24—N8—C28	119.8 (2)	C21—C20—C19	119.6 (3)
C24—N8—Pd1	120.24 (14)	C21—C20—H20	120.2
C28—N8—Pd1	119.90 (16)	C19—C20—H20	120.2
N1—C1—C2	121.4 (2)	C22—C21—C20	119.5 (2)
N1—C1—C5	113.28 (19)	C22—C21—H21	120.3
C2—C1—C5	125.07 (19)	C20—C21—H21	120.3
N2—C2—C1	120.8 (2)	C21—C22—C23	119.0 (2)
N2—C2—C10	113.82 (18)	C21—C22—H22	120.5
C1—C2—C10	125.21 (19)	C23—C22—H22	120.5
N2—C3—C4	122.2 (2)	N7—C23—C22	122.0 (2)
N2—C3—H3	118.9	N7—C23—H23	119.0
C4—C3—H3	118.9	C22—C23—H23	119.0
N1—C4—C3	121.7 (2)	N8—C24—C25	120.7 (2)
N1—C4—H4	119.2	N8—C24—C16	119.67 (19)
C3—C4—H4	119.2	C25—C24—C16	119.6 (2)
N3—C5—C6	121.3 (2)	C24—C25—C26	119.7 (2)
N3—C5—C1	119.16 (19)	C24—C25—H25	120.2
C6—C5—C1	119.5 (2)	C26—C25—H25	120.2
C5—C6—C7	119.1 (3)	C27—C26—C25	119.1 (2)
C5—C6—H6	120.4	C27—C26—H26	120.4
C7—C6—H6	120.4	C25—C26—H26	120.4
C8—C7—C6	119.2 (3)	C26—C27—C28	119.3 (2)
C8—C7—H7	120.4	C26—C27—H27	120.3
C6—C7—H7	120.4	C28—C27—H27	120.3
C7—C8—C9	119.3 (3)	N8—C28—C27	121.3 (2)
C7—C8—H8	120.4	N8—C28—H28	119.3
C9—C8—H8	120.4	C27—C28—H28	119.3
N3—C9—C8	122.1 (3)	O3—N9—O2	120.7 (3)
N3—C9—H9	119.0	O3—N9—O1	116.2 (3)
C8—C9—H9	119.0	O2—N9—O1	122.9 (3)
N4—C10—C11	120.8 (2)	O5—N10—O4	121.2 (3)
N4—C10—C2	119.52 (18)	O5—N10—O6	120.2 (3)
C11—C10—C2	119.6 (2)	O4—N10—O6	118.6 (3)
C12—C11—C10	119.8 (2)	C30—C29—H29A	109.5
C12—C11—H11	120.1	C30—C29—H29B	109.5
C10—C11—H11	120.1	H29A—C29—H29B	109.5
C13—C12—C11	119.1 (2)	C30—C29—H29C	109.5
C13—C12—H12	120.4	H29A—C29—H29C	109.5
C11—C12—H12	120.4	H29B—C29—H29C	109.5
C12—C13—C14	119.1 (2)	N11—C30—C29	179.9 (5)
C12—C13—H13	120.4		
			
C4—N1—C1—C2	−0.6 (3)	C18—N5—C15—C16	2.3 (3)
C4—N1—C1—C5	−175.51 (19)	C18—N5—C15—C19	176.1 (2)
C3—N2—C2—C1	3.9 (3)	C17—N6—C16—C15	−3.0 (3)
C3—N2—C2—C10	179.72 (19)	C17—N6—C16—C24	−177.0 (2)
N1—C1—C2—N2	−3.6 (3)	N5—C15—C16—N6	0.8 (3)
C5—C1—C2—N2	170.7 (2)	C19—C15—C16—N6	−172.3 (2)
N1—C1—C2—C10	−178.84 (19)	N5—C15—C16—C24	174.0 (2)
C5—C1—C2—C10	−4.5 (3)	C19—C15—C16—C24	1.0 (3)
C2—N2—C3—C4	−0.4 (3)	C16—N6—C17—C18	2.1 (4)
C1—N1—C4—C3	4.1 (3)	C15—N5—C18—C17	−3.2 (4)
N2—C3—C4—N1	−3.8 (4)	N6—C17—C18—N5	1.0 (4)
C9—N3—C5—C6	2.2 (3)	C23—N7—C19—C20	−2.4 (3)
Pd1—N3—C5—C6	−177.06 (17)	Pd1—N7—C19—C20	179.11 (16)
C9—N3—C5—C1	179.91 (19)	C23—N7—C19—C15	177.89 (19)
Pd1—N3—C5—C1	0.7 (3)	Pd1—N7—C19—C15	−0.6 (3)
N1—C1—C5—N3	−127.1 (2)	N5—C15—C19—N7	131.2 (2)
C2—C1—C5—N3	58.2 (3)	C16—C15—C19—N7	−55.3 (3)
N1—C1—C5—C6	50.6 (3)	N5—C15—C19—C20	−48.5 (3)
C2—C1—C5—C6	−124.1 (2)	C16—C15—C19—C20	125.0 (2)
N3—C5—C6—C7	−2.3 (3)	N7—C19—C20—C21	2.7 (3)
C1—C5—C6—C7	179.9 (2)	C15—C19—C20—C21	−177.6 (2)
C5—C6—C7—C8	0.5 (4)	C19—C20—C21—C22	−1.1 (4)
C6—C7—C8—C9	1.3 (4)	C20—C21—C22—C23	−0.6 (4)
C5—N3—C9—C8	−0.2 (3)	C19—N7—C23—C22	0.6 (3)
Pd1—N3—C9—C8	179.01 (18)	Pd1—N7—C23—C22	179.05 (17)
C7—C8—C9—N3	−1.6 (4)	C21—C22—C23—N7	0.9 (4)
C14—N4—C10—C11	0.1 (3)	C28—N8—C24—C25	2.4 (3)
Pd1—N4—C10—C11	177.58 (16)	Pd1—N8—C24—C25	−174.04 (17)
C14—N4—C10—C2	−177.47 (19)	C28—N8—C24—C16	−178.37 (19)
Pd1—N4—C10—C2	0.0 (3)	Pd1—N8—C24—C16	5.2 (3)
N2—C2—C10—N4	131.5 (2)	N6—C16—C24—N8	−134.9 (2)
C1—C2—C10—N4	−52.9 (3)	C15—C16—C24—N8	51.4 (3)
N2—C2—C10—C11	−46.0 (3)	N6—C16—C24—C25	44.3 (3)
C1—C2—C10—C11	129.5 (2)	C15—C16—C24—C25	−129.4 (2)
N4—C10—C11—C12	−1.5 (3)	N8—C24—C25—C26	−0.9 (4)
C2—C10—C11—C12	176.1 (2)	C16—C24—C25—C26	179.9 (2)
C10—C11—C12—C13	1.1 (4)	C24—C25—C26—C27	−0.8 (4)
C11—C12—C13—C14	0.5 (4)	C25—C26—C27—C28	0.8 (4)
C10—N4—C14—C13	1.7 (3)	C24—N8—C28—C27	−2.4 (3)
Pd1—N4—C14—C13	−175.86 (17)	Pd1—N8—C28—C27	174.09 (18)
C12—C13—C14—N4	−2.0 (3)	C26—C27—C28—N8	0.8 (4)

### Hydrogen-bond geometry (Å, º)

**Table:**

*D*—H···*A*	*D*—H	H···*A*	*D*···*A*	*D*—H···*A*
C3—H3···O4^i^	0.94	2.43	3.344 (3)	164
C4—H4···O1	0.94	2.51	3.330 (4)	146
C9—H9···O2^ii^	0.94	2.40	3.113 (4)	133
C14—H14···O5^iii^	0.94	2.43	3.257 (3)	147
C17—H17···O6^i^	0.94	2.43	3.343 (3)	164
C22—H22···O3^iv^	0.94	2.45	3.273 (5)	146
C23—H23···O2^ii^	0.94	2.54	3.275 (4)	136
C29—H29*A*···O1	0.97	2.58	3.405 (5)	144
C29—H29*C*···O4	0.97	2.51	3.459 (4)	167

Symmetry codes: (i) *x*+1, *y*, *z*; (ii) *x*, *y*+1, *z*; (iii) *x*+1, *y*+1, *z*; (iv) −*x*+1, −*y*+1, −*z*.
